# Effects of a Sudden Drop in Salinity on Immune Response Mechanisms of *Anadara kagoshimensis*

**DOI:** 10.3390/ijms20184365

**Published:** 2019-09-05

**Authors:** Mo Zhang, Li Li, Ying Liu, Xiaolong Gao

**Affiliations:** 1 Key Laboratory of Experimental Marine Biology, Institute of Oceanology, Chinese Academy of Sciences, Qingdao 266071, China (M.Z.) (Y.L.); 2 Marine Biology Institute of Shandong Province, Qingdao 266104, China; 3 College of Marine Technology and Environment, Dalian Ocean University, Dalian 116023, China; 4 State Key Laboratory of Marine Environmental Science, College of Ocean and Earth Sciences, Xiamen University, Xiamen 361102, China

**Keywords:** *Anadara kagoshimensis*, salinity, transcriptomics, cellular immunity, real-time quantitative PCR

## Abstract

In this experiment, the effects of a sudden drop of salinity on the immune response mechanisms of the ark shell *Anadara kagoshimensis* were examined by simulating the sudden drop of salinity that occurs in seawater after a rainstorm. Additionally, the differentially expressed genes (DEGs) were identified using transcriptome sequencing. When the salinity dropped from 30‰ (S30) to 14‰ (S14), the phagocytic activity of blood lymphocytes, the O_2_^−^ levels produced from respiratory burst, the content of reactive oxygen species, and the activities of lysozymes and acid phosphatases increased significantly, whereas the total count of blood lymphocytes did not increase. Total count of blood lymphocytes in 22‰ salinity (S22) was significantly higher than that in any other group. The raw data obtained from sequencing were processed with Trimmomatic (Version 0.36). The expression levels of unigenes were calculated using transcripts per million (TPM) based on the effects of sequencing depth, gene length, and sample on reads. Differential expression analysis was performed using DESeq (Version 1.12.4). Transcriptome sequencing revealed 269 (101 up-regulated, 168 down-regulated), 326 (246 up-regulated, 80 down-regulated), and 185 (132 up-regulated, 53 down-regulated) significant DEGs from comparison of the S14 vs. S22, S22 vs. S30, and S14 vs. S30 groups, respectively. Gene Ontology enrichment analysis of the DEGs in these salinity comparison groups revealed that the cellular amino acid metabolic process, the regulation of protein processing, the regulation of response to stress, and other terms were significantly enriched. Kyoto Encyclopedia of Genes and Genomes enrichment analysis showed that nucleotide-binding, oligomerization domain (NOD)-like receptor signaling pathway (ko04621), apoptosis-multiple species (ko04215), Toll and Imd signaling pathway (ko04624), NF-κB signaling pathway (ko04064), apoptosis (ko04210), and focal adhesion (ko04510) were significantly enriched in all salinity comparison groups. qRT-PCR verification of 12 DEGs in the above six pathways was conducted, and the results were consistent with the transcriptome sequencing results in terms of up-regulation and down-regulation, which illustrates that the transcriptome sequencing data are credible. These results were used to preliminarily explore the effects of a sudden drop of salinity on blood physiological and biochemical indexes and immunoregulatory mechanisms of *A.* *kagoshimensis*. They also provide a theoretical basis for the selection of bottom areas optimal for release and proliferation of *A.* *kagoshimensis* required to restore the declining populations of this species.

## 1. Introduction

The marine environment is being subjected to drastic changes due to global warming. Over the past 10–15 years, glaciers and ice sheets have disappeared at a fast rate accompanied by heavy rainfall [1]. The large inflow of fresh water affects different areas in the ocean in different ways. The salinity of surface seawater and nearshore water is prone to a substantial decrease during the rainy season, resulting in massive mortality and changes in the distribution area of marine organisms [2]. 

Salinity is a critical ecological factor in marine ecosystems, as it affects the metabolism, the osmotic adjustment, and the biological rhythm of marine organisms, and in turn it affects their distribution in nature [3,4]. Due to the effects of tide, evaporation, and seasonal rainfall, the salinity of seawater normally exhibits cyclic changes, and marine organisms have to adapt their own physiological activities to such changes of salinity [5,6,7]. Osmotic regulation by marine invertebrates is similar to that of vertebrates, and it includes perception, signal transduction, and physiological response. However, the physiological response of marine invertebrates is quite different from that of homoiosmotic animals [8]. 

The ark shell *Anadara kagoshimensis* is an important marine economic shellfish species in China. It is vertically distributed in the sea area between the low tide line and 7 m depth. Most individuals live in the fresh water-affected inner bay and neritic region; they exhibit a wide range of adaptation to salinity and prefer to inhabit the soft mud or the muddy seafloor with sand content [9]. Recently, overfishing and environmental changes have led to a drastic decrease of available resources. In order to restore the declining resources of *A.* *kagoshimensis*, large-scale enhancement and release of seeds have been conducted in some locations.

However, estuaries and intertidal zones are subjected to drastic changes in salinity, which can negatively impact organisms that live in the mud, are fixed to a substrate, or move slowly. Such organisms can grow well within an appropriate range of salinity, but deviation from their appropriate habitat salinity range can retard growth of the population and even lead to a stress response, which in turn can diminish immune resistance [10,11]. Therefore, to mediate the declining populations of *A.* *kagoshimensis*, it is necessary to develop a method to identify optimal seed release areas based on the tolerance mechanisms of this species to low salinity. 

The emergence of the G2 high-throughput sequencing technique has led to great discoveries in genome and transcriptome studies and enabled high-throughput, large-scale sequencing trials at an affordable cost. Marine shellfish can adapt to osmotic regulation by regulating RNA synthesis, protein metabolism, enzyme metabolism, ion transport, and cell volume, and intracellular free amino acids act as the primary osmoregulatory regulators [12]. Hosoi et al. found that amino acids, mainly taurine, played a crucial role in osmotic regulation by shellfish; in particular, the taurine transporter as a transport protein played a role in regulating hypotonic and hyperosmotic stress [13]. Additionally, the role of amino acid transporters and divalent cations in the osmotic regulation of shellfish has been reported [14,15,16]. Zhao et al. identified 48 differentially expressed genes (DEGs) from the *Crassostrea gigas* low-salinity stress group using the transcriptome analysis. Through annotation and enrichment analysis using Gene Ontology (GO) and Kyoto Encyclopedia of Genes and Genomes (KEGG), they found that most of these genes were related to the process of transcription, gene expression, and macromolecular biosynthesis and involved the taurine, the hypotaurine metabolic pathway, and the Cyclic Adenosine monophosphate (cAMP) signaling pathway [17]. Wang et al. performed high-throughput sequencing of the gill, the intestine, and the kidney of *Takifugu obscurus* cultured under different salinity levels using the Illumina G2 sequencing platform, and they found that, as the environmental salinity level increased, the cAMP signaling pathway was significantly down-regulated, while the thyroid hormones signaling pathway and the tricarboxylic acid cycle (TAC) cycle pathway were significantly up-regulated [18]. Zhang et al. explored how *Litopenaeus vannamei* can adapt to low salinity using transcriptome sequencing and reported that many DEGs were related to substance metabolism, energy metabolism, ion transport, and signal transduction, and results of KEGG analysis showed that ribosome, proximal tubulebicarbonate reclamation, fructose and mannose metabolism, and glutathione metabolism were significantly enriched [19]. 

This study was designed to identify the effects of a sudden drop of salinity on the immune response mechanisms of *A.* *kagoshimensis* cultured at different salinity levels and exposed to a sudden drop of salinity that mimicked conditions after a rainstorm. Immune parameters (phagocytic activity of blood lymphocytes, O_2_^−^ levels produced from respiratory burst, content of reactive oxygen species, activities of lysozymes and acid phosphatases, and total count of blood lymphocytes) were measured. We also compared and analyzed the variation of osmotic regulation-related genes in *A.* *Kagoshimensis* cultured at different salinity levels using the transcriptome technique and screened for DEGs and key signaling pathways in individuals exposed to a sudden drop of salinity. The goal was to provide a scientific basis for understanding the physiological adaptation process of marine invertebrates exposed to low salinity. The results also can be used to develop a management plan for enhancement and release of seeds to restore *A.* *kagoshimensis* populations. 

## 2. Results

### 2.1. Total Count of Blood Lymphocytes and Phagocytic Activity

When the salinity decreased from 30‰ to 22‰, total count of blood lymphocytes in *A.* *kagoshimensis* increased significantly (*p* < 0.05, Table 1). However, as the salinity continued to drop to 14‰, total count of blood lymphocytes did not continue to increase; in contrast, the value was significantly lower than that in the S22 group (*F* = 111.302, *df1* = 2, *df2* = 24, *p* < 0.01). With the sudden drop of salinity, the phagocytic activity of blood lymphocytes was significantly enhanced, and the phagocytic activity of blood lymphocytes in S14 was significantly higher than that in any other group (*p* < 0.001).

### 2.2. O_2_^−^ Levels Produced from Respiratory Burst of Blood Lymphocytes and ROS Content

No significant difference in O_2_^−^ levels produced from respiratory burst of blood lymphocytes was detected between S30 and S22, but the value for each of these two groups was significantly lower than that in S14 (*F* = 149.281, *df1* = 2, *df2* = 24, *p* < 0.01, Table 1). No significant difference in reactive oxygen species (ROS) content was identified between S30 and S22, but the value for each of these two groups was significantly lower than that in S14 (*p* < 0.001). 

### 2.3. Lysozyme (LZM) and Acid Phosphatase (ACP) Activities

**Figure 1:**
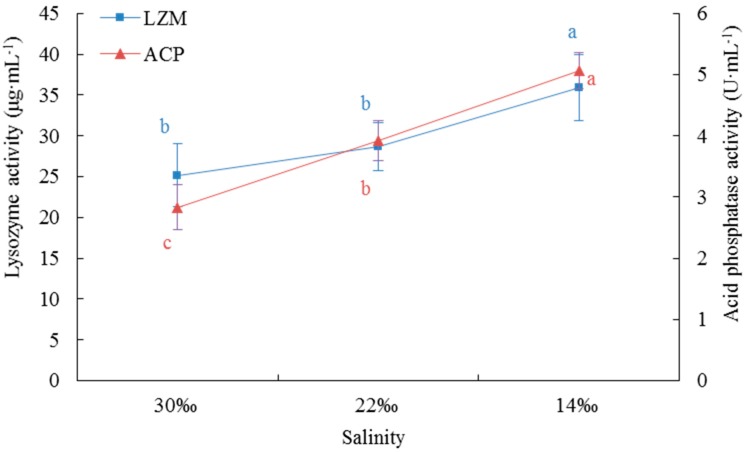
Effects of a sudden drop in salinity on the activities of lysozyme (LZM) and acid phosphatase (ACP) in *Anadara kagoshimensis*. Values are expressed as mean ± SE (*n* = 9). Statistical analysis was performed by one-way ANOVA followed by Tukey’s test using SPSS version 18.0. Means with different lower case letters are significantly different at *p* < 0.05 level.

The activity of lysozyme (LZM) in *A.* *kagoshimensis* in S14 was significantly higher than that in S22 or S30, but the activity of LZM did not differ significantly between S22 and S30 (*p* > 0.05, Figure 1). The activity of acid phosphatase (ACP) increased significantly with the sudden drop of salinity, as the activity of ACP in *A.* *kagoshimensis* in S14 was significantly higher than that in S30 and S22 (*F* = 202.467, *df1* = 2, *df2* = 24, *p* < 0.001, Figure 1). 

### 2.4. Transcriptome Sequencing and Splicing Assembly

Transcriptome sequencing was performed on *A.* *kagoshimensis* specimens from the three different salinity groups using Illumina Hi-seq 2500, and the sequences with 392,231,758 original reads were constructed (NCBI No. PRJNA548266). The gene sequencing and assembly results are shown in Table 2. After low-quality sequences were removed, 381,773,678 high-quality reads (97.33% of total reads) were obtained. In addition, 480,426 transcripts were spliced using high-quality reads, with the length ranging from 201 to 16,628 base pairs (bp) and an average size of 716 bp. The longest transcript of each gene was used as the unigene. In total, 194,861 pieces of unigenes were identified with an average size of 644 bp. 

### 2.5. Functional Annotation of Transcriptome Sequences

All 194,861 unigene sequences were compared through the Blast X to NCBI non-redundant protein (NR), NCBI non-redundant nucleotide sequences (NT), eukaryotic orthologous groups (KOG), conserved domain database (CDD), PFAM, Swissprot, TrEMBL, GO, and KEGG databases. The sequence information that was highly similar to the given unigene sequence was used to obtain the connotation information. From this analysis, 3000 (1.54%) unigenes were annotated from nine databases (Table 3). 

### 2.6. Functional Classification Based on GO and KEGG Analysis

Using the GO database, 33,522 unigenes were annotated, which then were divided into three functional groups: cellular component (CC), molecular function (MF), and biological process (BP) (Supplementary Figure S1A). In the BP classification, most genes were enriched under cellular process (GO: 0009987) at 67.27% of total genes. In the CC classification, most genes were enriched under cell (GO: 0005623) and cell part (GO: 0044464) at 73.59% and 73.47%, respectively. In the MF classification, most genes were enriched under binding (GO: 0005488) and catalytic activity (GO: 0003824) at 61.65% and 41.89%, respectively. 

A total of 19,868 unigenes in 26 subtypes were successfully annotated and classified in the KOG database. “Signal transduction mechanisms” was the subtype with the highest percentage of annotated unigenes at about 19.01%. This category was followed by “General function prediction” only and “Posttranslational modification, protein turnover, chaperones” at 12.84% and 10.93%, respectively (Supplementary Figure S1B).

The KEGG database was used to construct the appropriate pathway for the unigene sequences such that 12,644 unigenes were divided into five biochemical pathway categories, including “Organismal Systems” (6879), “Metabolism” (6771), “Genetic Information Processing” (3517), “Environmental Information Processing” (4085), and “Cellular Processes” (3988). Of these KEGG categories, the subcategories with the largest proportions were “Endocrine system” (1588, 23.09%), “Carbohydrate metabolism” (1107, 16.35%), “Translation” (1519, 43.19%), “Signal transduction” (3093, 75.72%), and “Transport and catabolism” (1530, 38.37%) (Supplementary Figure S1C).

### 2.7. Screening of DEGs and GO and KEGG Pathway Enrichment Analysis

**Figure 2:**
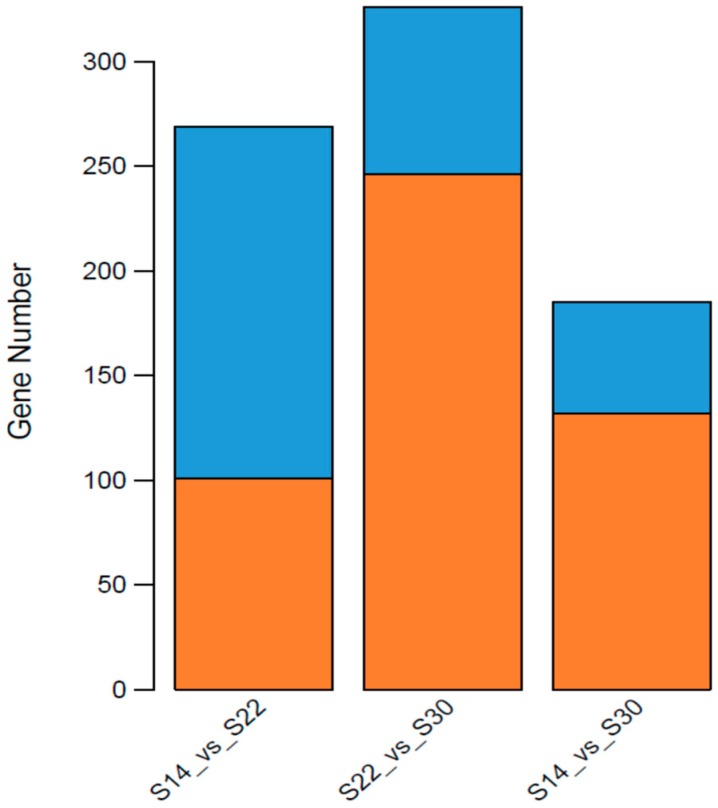
Number of differentially expressed genes (DEGs) of *Anadara kagoshimensis* among different salinity treatments. The x-axis represents the pairwise comparisons, and the y-axis shows the number of DEGs screened. Orange bars denote up-regulated genes, and blue bars indicate down-regulated genes.

**Figure 3:**
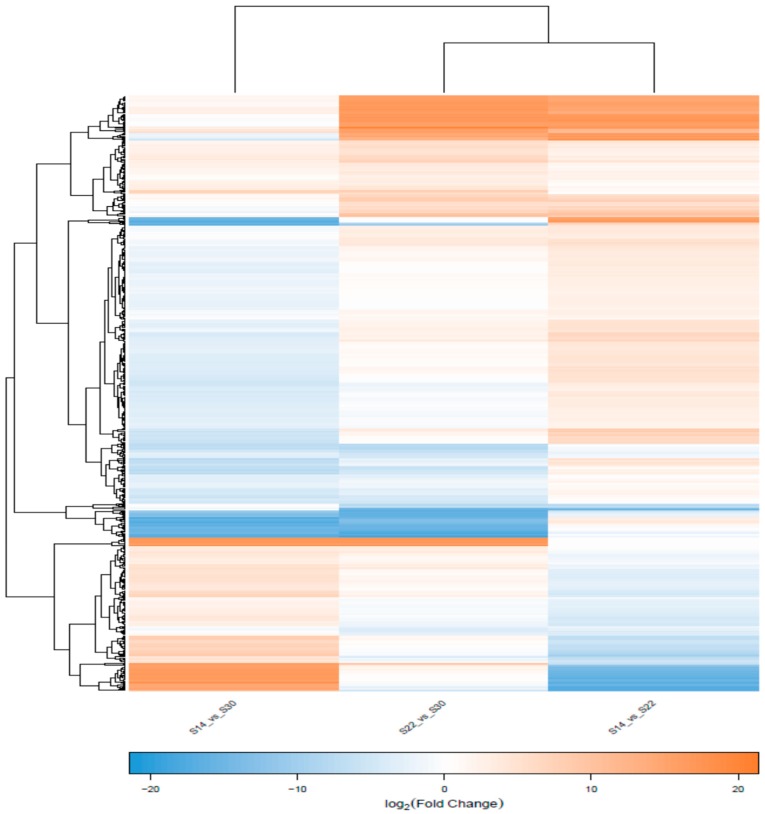
Hierarchical clustering of DEGs in the three groups of *Anadara kagoshimensis* with different salinity treatments. The intensity of the color from green to red indicates the magnitude of differential expression. Orange and blue indicate up- and down-regulation, respectively.

Comparing the gene expression between the three salinity groups revealed that the number of DEGs in S14 vs. S22 was 269 (101 up-regulated, 168 down-regulated), S22 vs. S30 was 326 (246 up-regulated, 80 down-regulated), and S14 vs. S30 was 185 (132 up-regulated, 53 down-regulated) (Table 4, Figure 2). Expression pattern clustering analysis of significant DEGs was performed to find common expression points between different genes, and nine specimens were divided into three distinctive groups according to the salinity differences (Figure 3).

GO enrichment analysis performed on DEGs in S14 vs. S22, S22 vs. S30, and S14 vs. S30 groups showed that 78 (17 up-regulated, 61 down-regulated), 98 (78 up-regulated, 20 down-regulated), and 32 (20 up-regulated, 12 down-regulated) DEGs were enriched in GO terms. Further analysis of the significantly enriched GO terms in “Biological Process” revealed the results given in Table 5: in S14 vs. S22, 18 biosynthesis and metabolism related processes were significantly enriched (corrected *p* < 0.05); in S14 vs. S30, no process was significantly enriched (corrected *p* < 0.05); in S22 vs. S30, 21 protein processing and catabolism related processes were significantly enriched (corrected *p* < 0.05).

**Figure 4:**
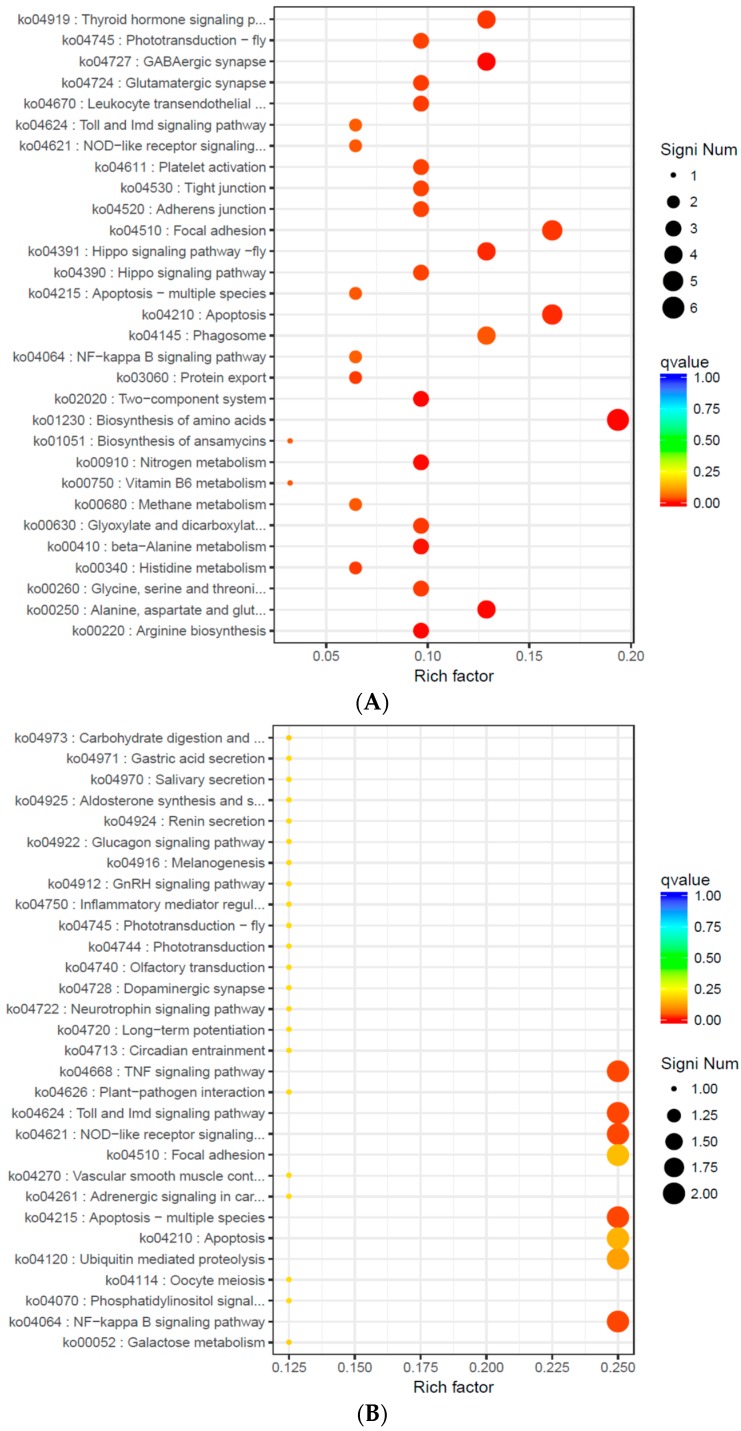

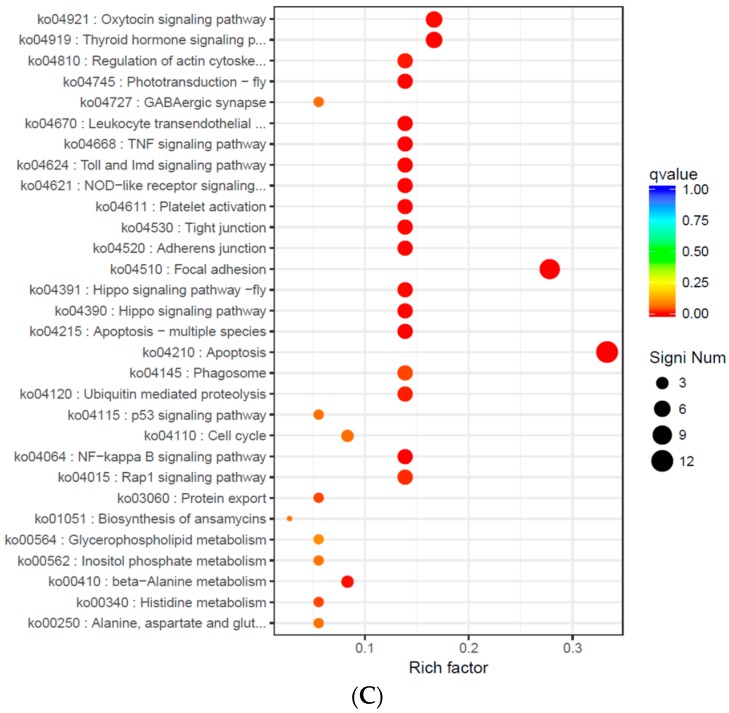
Scatterplot of KEGG pathways enriched in the DEGs for each pairwise comparison [(**A**) S14 vs S22; (**B**) S14 vs S30; (**C**) S22 vs S30]. “Rich factor” represents the ratio of DEG numbers annotated with a pathway term relative to all genes annotated with this pathway term. A higher rich factor indicates greater intensity. The qvalue is the corrected *p* value (range, 0 to 1), and a lower *q* value denotes greater intensity. Only the top 30 enriched pathway terms are shown.

To identify the pathway that was more significantly enriched in the significant DEGs compared with the entire genomic background, KEGG pathway enrichment analysis was performed using the hypergeometric test. DEGs in S14 vs. S22, S22 vs. S30, and S14 vs. S30 were enriched in 30, 29, and nine pathways (*p* < 0.05, Figure 4), respectively. In all comparison groups, NOD-like receptor signaling pathway (ko04621), apoptosis-multiple species (ko04215), Toll and Imd signaling pathway (ko04624), NF-κB signaling pathway (ko04064), apoptosis (ko04210), and focal adhesion (ko04510)” were significantly enriched. In S14 vs. S22 and S22 vs. S30, GABAergic synapse (ko04727), alanine, aspartate, and glutamate metabolism (ko00250), thyroid hormone signaling pathway (ko04919), and histidine metabolism (ko00340) were also significantly enriched (Figure 4).

### 2.8. qRT-PCR Verification

**Figure 5:**
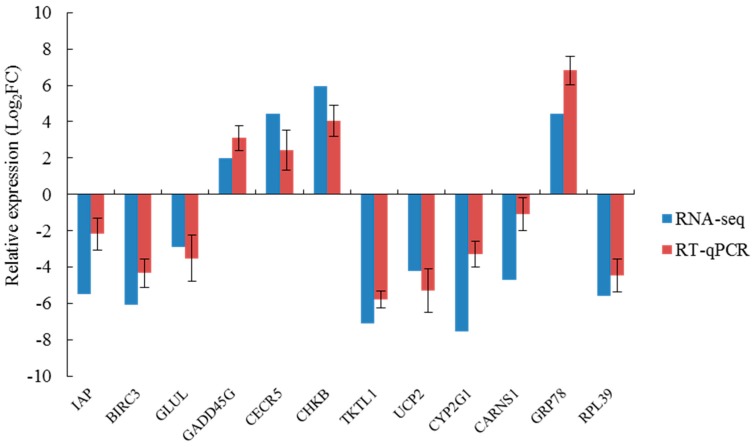
Genes selected for real-time PCR validation and the fold change in three salinity treatments for both RNA-seq and RT-PCR analysis. *IAP*, inhibitor of apoptosis protein; *BIRC3*, baculoviral IAP repeat-containing protein 3; *GLUL*, glutamine synthetase; *GADD45G*, growth arrest and DNA damage-inducible protein; *CECR5*, cat eye syndrome critical region protein 5; *CHKB*, choline/ethanolamine kinase; *TKTL1*, transketolase-like protein 1; *UCP2*, mitochondrial uncoupling protein 2; *CYP2G1*, cytochrome P450 2G1; *CARNS1*, carnosine synthase 1; *GRP78*, glucose-regulated protein; *RPL39*, ribosomal protein L39. The relative expression values were normalized to β-actin gene expression. Vertical bars represent the mean ± S.D. (*n* = 3).

According to the KEGG significant enrichment analysis of DEGs in each salinity group as described above, 12 key DEGs related to amino acid synthesis and metabolism, apoptosis, and immunity were identified, and they fell into six significantly enriched signaling pathways. Table 6 gives the corresponding primer sequences. Transcripts per million (TPM) values of individual groups obtained from the transcriptome were verified with qRT-PCR. Although the qRT-PCR verification results and the mRNA sequencing results differed in some ways or in some cases, they had the same trend of up-regulation and down-regulation (Figure 5). In all comparison groups, the expression levels of *IAP*, *BIRC3*, *GLUL*, *TKTL1*, *UCP2*, *CYP2G1*, *CARNS1*, and *RPL39* tended to decrease significantly (*p* < 0.05). In contrast, the expression levels of *GADD45G*, *CECR5*, *CHKB*, and *GRP78* tended to increase significantly (*p* < 0.05). Thus, the comparison results verified the consistency between transcriptome sequencing and qRT-PCR results. We also checked for DEGs in *A. kagoshimensis* that were acclimated at 30‰ and then moved to the 30‰ test tank, as shown in Supplementary Figure S2. Expression levels of the 12 DEGs before and after specimens were moved to the 30‰ salinity treatment were not significantly different (*p* > 0.05). This result confirms that the DEGs reported herein were due to salinity changes and not to differences in *A. kagoshimensis* handling.

## 3. Discussion

Salinity is a limiting factor in the survival and the distribution of many marine organisms, and decreased salinity can lead to massive deaths and migration of species [20,21]. Marine shellfish lack lymphocyte-based specific immune function, thus immune defense mainly depends on phagocytosis, encapsulation, and other functions of blood cells as well as regulation of various non-specific immune factors in body fluids, and blood cells are vulnerable to the impact of salinity [22]. Hooper et al. suggested that the temporary decrease in the number of blood lymphocytes of “withering”-affected *Haliotis cracherodii* can be attributed to apoptosis and aging of blood lymphocytes in the hemolymph [23]. *Vibrio anguillarum* stimulation and hypoxia treatment both had a significant impact on the expression of hemoglobin, which first increased after treatment and then decreased. Hemoglobin plays a significant role in the process of bacterial and anoxia tolerance reaction in *Scapharca broughtonii,* which suggests that hemoglobin is a key immune factor [24]. Marine invertebrates primarily respond to changes in salinity in the environment by regulating osmotic pressure of the hemolymph. When the salinity decreased from 30‰ to 22‰ in our study, total count of lymphocytes in *A.* *kagoshimensis* significantly increased. This finding indicates that increasing the amount of blood lymphocytes is an effective means for the organism to cope with environmental stress. However, when the salinity dropped to 14‰, the blood lymphocyte count did not increase further but was instead significantly lower than that at 22‰. Thus, the wide range of the sudden drop of salinity presumably caused drastic changes in osmotic pressure during the passive water absorption process. This process involves continuous transmembrane transport of inorganic ions, which can cause damage to the cell membrane structure and which may eventually induce cell rupture and death. 

The phagocytic activity of blood lymphocytes is a critical indicator of the defense function of blood lymphocytes [25,26]. In this study, the phagocytic activity of lymphocytes significantly increased with the sudden drop of salinity, indicating that *A.* *kagoshimensis* adapted to salinity changes by increasing the intensity of its immune response. ROS produced from respiratory burst is a critical mechanism that enables phagocytic cells to kill heterologous organisms. Respiratory burst may occur during phagocytosis of blood lymphocytes, and ROS produced by respiratory burst is an important immune defense factor in organisms [27,28]. As the salinity decreased in this study, the O_2_^−^ levels and the ROS content produced from respiratory burst of blood lymphocytes showed a similar trend. The ROS content in S14 was significantly higher than that in any other group, indicating that the bactericidal activity of leukocytes and the host congenital cellular immune response was enhanced as the ROS content increased at lower salinity. This process would eventually improve the survival rate of the organism in response to environmental stress.

Cellular immunity and humoral immunity are closely linked, and endogenous humoral immune factors also enhance cell migration and phagocytosis [29,30]. LZM is a basic humoral immune factor and constitutes an integral part of humoral immunity in invertebrates [31,32]. A 1 mL suspension containing 1 × 10^9^ *Vibrio anguillarum* was rejected in the foot of *S. broughtonii*, and the content of LZM in the blood rapidly increased, whereas the content of LZM in the gill and the foot was significantly higher than that in the control group 24 h after the injection, indicating that blood is one of the primary immune tissues of *S. broughtonii* [33]. The activity of LZM was not significantly different between S30 and S220 in this study, but when the salinity further decreased to 14‰, LZM activity was significantly higher than that in the higher salinity groups. This enhancement of activity of this critical humoral immune factor was an effective way to improve the antibacterial ability and the non-specific immunity of the organism under environmental stress. ACP is an integral component of lysosomes in the immune system. Pathogen invasion into the body of an animal stimulates the release of ACP via phagocytosis of phagocytes, and ACP exploits its immune function by hydrolyzing the phosphate esters on the surface of pathogenic bacteria [34,35]. With the sudden drop of salinity in our experiment, the phagocytic activity of blood lymphocytes and the corresponding ACP activity significantly increased. This result suggests that *A.* *kagoshimensis* can effectively resist infection by external pathogens in response to environmental stress via identification, digestion, degradation, and endocytosis of foreign bodies.

After the low-quality sequences were removed, 381,773,678 high-quality reads (97.33% of total reads) were obtained, which is a result superior to that for other molluscs (e.g., 95.3% for *Crassostrea angulate* [36], 94.07% for *Reishia clavigera* [37], and 96.95% for *Rapana venosa* [38]). Additionally, the number of unigenes obtained in our study (194,861) was significantly higher than that for other shellfish species assembled using 454 GSFlx sequencing (e.g., 139,397 for *Patinopecten yessoensis* [39] and 124,732 for *Meretrix* [40]). This indicates that the sequencing depth of the Hi-seq 2500 Sequencing Platform is significantly better than that of 454 GSFlx, and that it is able to identify unigenes with low expression levels. GO enrichment analysis of DEGs revealed that those in S14 vs. S22 were mainly enriched in biological process terms, including oxoacid metabolic process, organic acid metabolic process, carboxylic acid metabolic process, cellular amino acid metabolic process, and alpha-amino acid metabolic process. Those in the S22 vs. S30 comparison were mainly enriched in biological process terms, including protein maturation, regulation of protein processing, regulation of protein maturation, and protein processing. No term related to biological process was identified in the S14 vs. S30 comparison according to the corrected *p* value. However, if *p* < 0.05 was used as the only criterion, DEGs were found to be mainly enriched in biological process terms, including regulation of response to stress, protein modification by small protein conjugation or removal, regulation of catalytic activity, and proteolysis. Most of these DEGs were found to be closely related to metabolism, apoptosis, and the immune system. In S14 vs. S22, S22 vs. S30, and S14 vs. S30, genes related to amino acid metabolism, protein maturation, and response to stress played a dominant role in the response process of *A.* *kagoshimensis* to the sudden change of salinity. This suggests that, as the intensity of the sudden drop of salinity increased, the organism could improve its adaptability and its tolerance to the external environment by effectively adjusting its physiological processes.

Free amino acids in marine shellfish are another important osmotic effector, and they arise mainly from the decomposition of tissue cells or hemolymph protein. When shellfish are placed in a low-salinity environment, their cells swell due to the moisture produced from the osmotic pressure difference. Any change of plasma membrane permeability may induce the outflow of specific amino acids, and cells remove some excessive moisture by efflux of some low molecular weight free amino acids to restore the cell volume [41]. Taurine, glycine, and alanine are the most commonly used free amino acids, and changes in taurine content account for the vast majority of free amino acid changes [42]. In this study, alanine, aspartate, and glutamate metabolism and histidine metabolism were significantly enriched in S14 vs. S22 and S22 vs. S30. The pathways related to amino acid metabolism, including beta-alanine metabolism, glycine, serine, threonine metabolism as well as arginine biosynthesis, were also significantly enriched in S14 vs. S22. These findings suggest that arginine, glycine, and threonine play an important role in osmotic regulation of *A.* *kagoshimensis*. In these amino acid metabolic pathways, glutamine synthetase, 4-aminobutyrate aminotransferase, carnosine synthase 1, phosphoserine aminotransferase, betaine-homocysteine *S*-methyltransferase, and d-3-phosphoglycerate dehydrogenase tended to be significantly down-regulated after a sudden drop of salinity, which further confirmed that *A.* *kagoshimensis* could maintain the equilibrium of osmotic pressure by changing the concentration of free amino acids in cells under low salinity. For *C. gigas* under a low osmotic condition, most free amino acids in the mantle cells showed a significant, synchronous decrease within 2–8 h; under a high osmotic condition, the contents of glycine, alanine, and taurine significantly increased, and the rapid increase of alanine played a critical role in the short-term adaptation to salinity change while taurine acted in long-term adaptation [43]. Zhao et al. also reported that taurine plays a crucial role in the regulation of osmotic osmosis, taurine and subtaurine metabolic pathways serve as the main metabolic pathways for taurine production, and the cAMP signal transduction pathway might be involved in the signal transduction of external changes in osmotic pressure [17]. Further, McNamara et al. found that free amino acids can stabilize macromolecules, including proteins, better than inorganic ions without changing the structure and the function of enzymes or causing internal disorder [44]. Free amino acids not only serve as important precursors for protein synthesis, but they also are involved in oxidative metabolism to provide energy to the organism, serve as a critical component of substance transporters, and can mediate signal transmission as a neurotransmitter [45]. For example, glutamic acid and aspartic acid are neurotransmitters linked with excitatory conduction that can participate in metabolic regulation as a coenzyme component, and glycine and γ-aminobutyric acid are inhibitory transmitters [46,47]. Thus, free amino acids not only effectively regulate osmotic pressure, but they also enhance the signal transduction capabilities of the nervous system in mediating the immune response under environmental stress.

In S14 vs. S22 and S22 vs. S30, thyroid hormone signaling pathway was also significantly enriched; the expression levels of fibropellin-3 were significantly down-regulated, indicating that endocrine hormones were involved in the response of *A. kagoshimensis* to environmental stress. When subjected to environmental stress, *C. gigas* can regulate phagocytic activity, apoptosis index, and synthesis activity of immune-related enzymes by releasing catecholamine, thereby mediating the response of the organism to environmental stress. As the most important immune cell in *C. gigas*, blood lymphocytes can actively mediate neuroimmune regulation by changing the expression levels and the binding activity of its surface neurotransmitter receptors [48]. D-Ala2-D-Met^5^-Enkephalinamide may induce activation and directional migration of *Mytilus edulis* immune cells, accelerate the adhesion process of blood lymphocytes, and induce immune cells and ganglia to release interleukin-1, thereby enhancing the activity of immune cells [49]. As a result, molluscs respond to internal and external environmental stimuli with complex response activities that are under the joint regulation of the neuroendocrine and the immune systems [50]. Results of the current study confirm this with premise.

Apoptosis is the process of active cell death that is regulated by genes. Subject to certain signal stimuli, the apoptosis process is initiated, and the apoptotic cells eventually are phagocytosed by phagocytic cells [51]. In the three salinity comparison groups in this study, two KEGG enrichment pathways (Apoptosis-multiple species and Apoptosis) were significantly enriched. The expression levels of the *inhibitor of apoptosis protein* and *Baculoviral IAP repeat-containing protein 3* were significantly down-regulated, and those of *DNA damage-inducible protein* and *death-associated inhibitors of apoptosis 2* were significantly up-regulated, indicating that the apoptosis system plays an important role in shellfish immunity. IAPs is a type of protein that inhibits apoptosis action, participates in tumor necrosis factor receptor (TNFR)-mediated signal transduction by inhibiting caspase, and interacts with NF-κB to exert antiapoptosis effects [52]. In the current study, the NF-κB signaling pathway was significantly enriched in each salinity comparison group, indicating that IPAs can interact with NF-κB to form a regulatory network to implement orderly control of apoptosis. During the immune response, apoptosis can remove unintended or potentially dangerous cells (such as virus-infected cells) and acts as the main defense mechanism. A high salinity environment resulted in apoptosis in blood lymphocytes of *Crassostrea virginica*, and a high temperature environment (28 °C) also induced apoptosis in blood lymphocytes of *C. virginica* [53,54]. Contaminants such as heavy metals, tributylbutenes, and polycyclic aromatic hydrocarbons can induce apoptosis in immune or non-immune cells of molluscs, and the effects are linked to concentration of contaminants and duration and method of application [55,56]. Russo et al. reported that *Lymnaea stagnalis* removes environmental contaminants such as herbicides and insecticides by initiating the apoptosis procedure of lymphocytes, and this reaction can be enhanced by producing ROS [57]. In response to a variety of environmental stimuli, ROS and oxidative stress in molluscs increase, which in turn causes oxidative damage to cells and induces apoptosis [58]. In our study, when the salinity decreased to 14‰, the ROS content significantly increased, suggesting that the oxidative damage caused by environmental stress would eventually induce apoptosis. In order to enhance the adaptability of an organism to environmental stress, the organism initiates the apoptosis procedure to selectively remove internal cells, which is of great significance for the stability of the normal cell population in tissues and the immune defense response.

## 4. Materials and Methods

### 4.1. Source and Acclimation of A. kagoshimensis 

*A.kagoshimensis* (shell length: 30.15 ± 1.46 mm, body weight: 7.97 ± 1.15 g) were purchased from Fuyuan fisheries company (Rizhao, Shandong, China), and all experimental *A.* *kagoshimensis* were sourced from the same batch after artificial hatching. After purchasing the abalone, they were acclimated in one culture container (length 1.2 m× width 1 m× height 1 m, water volume: 1200 L) for 15 d; water temperature was kept at 20 °C, salinity at 30 ± 1‰, pH at 7.9, dissolved oxygen concentration at >6 mg/L. The light emitting diode (LED) light was hung above the container, the light cycle was 14:10 light:dark, and lights were turned on at 06:00 h and off at 20:00 h via a clock controller. Aquaculture water was obtained from the natural sea area and used after sedimentation and sand filtration. Two-thirds of the water was replaced with fresh seawater each day at 09:00 to ensure good water quality. During the period of acclimation, the food mixture of *Chlorella vulgaris*, *Isochrysis galbana*, and *Platymonas subcordiformis* was fed once a day at a volume ratio of 1:1:1, then the food concentration was measured every 6 h.

All *A.* *kagoshimensis* in this study were handled in strict accordance with China legislation on scientific procedures on living animals. The protocol was approved by the ethics committee at University of Chinese Academy of Science (permit number: 399 20021109, 9 November 2002).

### 4.2. Experimental Design

In this experiment, three salinity groups were established. Seventy-two *A.* *kagoshimensis* individuals (three biological replicates × 8 *A.* *kagoshimensis* per biological replicate for each salinity group) that were cultured under normal seawater salinity of 30‰ were placed directly in seawater with salinity of 30‰ (S30), 22‰ (S22), or 14‰ (S14) for 72 h. The low-salinity seawater was prepared using tap water with 24 h aeration and seawater prepared through natural sand filtration. The S30 group served as the control. At the end of the experiment, nine randomly selected individuals from each treatment group were shelled, and the gill tissue was removed with scissors and tweezers, placed into 1.5 mL centrifuge tubes (three gills were combined into one sample), and immediately stored in liquid nitrogen for use in the transcriptomics and related gene expression assays. The adductor muscle from another three randomly selected individuals from each container (nine individuals in total at each treatment group) was removed, and the hemolymph was extracted from the sinusoids using a 10 mL medical syringe; these samples were used to determine the hematological indexes and the immunoenzymatic activity.

### 4.3. Assay of Total Count of Blood Lymphocytes and Phagocytic Activity

To measure the total count of blood lymphocytes and phagocytic activity, 50 μL of hemolymph liquid were fixed with the equivalent amount of 10% neutral formalin. After mixing the solution evenly, the number of blood cells was counted using a counting plate at 400× under an optical microscope (Qiujing Inc., Shanghai, China). The phagocytic activity analysis was conducted following the method described by Zhang et al. [59]. First, 100 μL of sterilized anticoagulant were added to 100 μL hemolymph. After evenly mixing the solution, a 50 μL cell suspension was coated on a glass slide, and 10–15 min later, the cells were adhered to the slide. Following attachment, 50 μL of yeast suspension (Baker’s yeast, Type II, Sigma, St. Louis, MO, USA) were added to the cell monolayer. They were washed carefully with sterilized phosphate buffered saline (PBS) twice, fixed with methanol after the serum was removed, stained with Giemsa, dried and restained with 1% methylene, and observed and counted under the oil immersion lens to calculate the phagocytic rate as follows:

Phagocytic rate = (Number of phagocytes/Total number of cells observed) × 100

### 4.4. Measurement of O_2_^−^ Levels Produced by the Respiratory Burst of Blood Lymphocytes

Three hundred µL of hemolymph from each ark shell were used for this analysis. The sample was injected into three ELISA plate wells (100 μL hemolymph per well). The samples were incubated at room temperature for 20 min, and the hemolymph liquid was discarded. The remaining sample was washed with 100 μL of PBS once, and 100 μL of 0.3% nitroblue tetrazolium (NBT) solution were added. The mixture was allowed to react at room temperature for 30 min, followed by centrifugation at 800× *g* at 4 °C for 10 min. The supernatant was discarded, and the remaining sample was fixed with 200 μL of anhydrous methanol, washed with 70% ethanol twice, and air dried. The precipitates were dissolved with 120 μL of 2 M potassium hydroxide and 140 μL of dimethyl sulfoxide. After even mixing, the optical density (OD) was measured using an ELISA reader (Bio-Rad Laboratories, Inc., Irvine, CA, USA) [60]. The respiratory burst levels of lymphocytes were expressed as the reduction amount of NBT produced per 100 μL of hemolymph (expressed in OD_630_).

### 4.5. ROS and Immune-Related Enzyme Activity Assays

Levels of reactive oxygen species, lysozyme, and acid phosphatase activities were measured using kits purchased from Nanjing Jiancheng Bioengineering Institute (Nanjing, China). First, 0.2–0.4 g of gill tissues were mixed with 1.8 mL of 0.86% saline, fully ground in an ice-water bath, then centrifuged at 3500 g/min for 10 min to prepare the 10% tissue homogenate for the measurement of LZM and ACP activities. To measure ROS content, the tissue homogenate was centrifuged at 1000× *g* for 10 min, and then 1 mmol/L DCFH-DA (2′,7′-dichlorofluorescin diacetate) was added to the supernatant. The mixture was incubated at 37 °C for 30 min after being fully and evenly mixed. A fluorescence spectrophotometer (721G-100, INESA CC, Shanghai, China) was used to determine the fluorescence intensity at the optimum excitation wavelength of 500 nm and at the optimum emission wavelength of 525 nm, and the results were expressed as fluorescence arbitrary units (A.U.) [61]. 

LZM activity was measured using the turbidimetric method [62]; the reaction substrate was 0.2 mg/mL *Micrococcus* (Sigma) suspension prepared in 0.05 mol/L, pH 6.1 phosphate buffer, and the absorbance was measured at 0.5 min and 4.5 min at 530 nm using a spectrophotometer. The activity of LZM was defined as the absorbance of the bacteria solution reduced by 0.001 per min (namely an active unit).

The activity of ACP was measured following the method described by Góth [63]; 500 μL of hemolymph were centrifuged at 1500× *g* at 4 °C for 10 min, the supernatant was discarded, and 100 μL of sterilized PBS were added to resuspend the blood cells (concentrated 5-fold). The sample then was centrifuged at 15,000× *g* at 4 °C for 5 min, and the supernatant was removed for testing. The activity of ACP was defined as the hemocyte dissolved matter in 100 mL of hemolymph reacted with the matrix solution (200 μL 4-aminoantipyrine, 400 μL potassium ferricyanide) at 27 °C for 15 min to produce 1 mg phenol (namely an active unit).

The protein content in the homogenate was measured using Coomassie blue staining, as described by Bradford [64], and bovine serum albumin was used as the protein marker.

### 4.6. RNA Extraction, cDNA Library Construction, and Transcriptome Sequencing

Each treatment had 3 replicates for RNA extraction, and each replicate contained 3 individuals of *A. kagoshimensis*. Before RNA extraction of each replicate, the samples of the 3 individuals were mixed together. Total RNA was extracted from the gill sample of 3 treatment groups with Trizol Reagent (Invitrogen, Carlsbad, CA, USA). The quality of total RNA was detected with a Bioanalyzer 2100 (Agilent, CA, USA), and the concentration of total RNA was detected with RNA 6000 Nano LabChip Kit (Agilent, CA, USA). According to the requirements of transcriptome sequencing, the total amount of RNA per library construction was 5 ug with the concentration of RNA ≥ 200 ng/μL. The OD260/280 was in 1.8~2.2. The kit used for library construction was the Illumina TruSeq RNA sample Preparation Kit (Illumina, San Diego, CA, USA). After the total RNA extracted from the samples was tested to be qualified, mRNA from the magnetic bead enriched eukaryote connected with Oligo (dT) was used. Next, 5 ug of total RNA was extracted from each sample for construction of a cDNA library. The extracted mRNA was randomly broken by Beads Wash Buffer into short fragments. The fragmented mRNA was used as a template, followed by synthesis of cDNA with a six-base random primer (Random hexamers). Next, dNTPs, RNaseH, and DNA polymerase were added for synthesis of the second strand. The double-stranded product was purified with AMPure XPbeads. The cohesive end of the DNA was repaired using the activity of T4 DNA polymerase and Klenow DNA polymerase as the blunt end. The 3’ end was added with adenine and a linker. The fragments were selected with AMPure XPbeads. PCR amplification was ultimately made to obtain the final sequencing libraries. After the libraries were tested to be qualified, the paired-end sequencing was performed using Illumina Hiseq 2500 (Illumina, San Diego, CA, USA).

#### 4.6.1. Quality Control of Transcriptome Sequencing Data and Trinity Splicing

The raw data obtained from sequencing were processed with Trimmomatic (Version 0.36) [65]. Clean reads were acquired by removing low-quality reads, sequences with N ratio >5%, and adapter sequences in reads. Ten thousand pieces of sequences were extracted from these clean data to run a BLASTN comparison with the NCBI NT database; results with e-value ≤ 1 × 10^−10^, similarity >90%, and coverage >80% were used to evaluate the species distribution and to detect contamination. The clean data, de novo, were assembled into a transcript using Trinity (https://github.com/trinityrnaseq/trinityrnaseq/wiki, Version 2.4.0) with min_kmer_cov 2 as the parameter [66]. The Trinity splicing process involves three steps: first, assemble reads of RNA-seq into a unique sequence; second, cluster contigs generated in the first step, then construct the Bruijn graph for each cluster; last, process the Bruijn graphs, search for a path according to reads and paired reads, as shown in the graph, until full-length transcripts with alternative splicing are obtained, and separate paralogs. The next step involves removing redundancy of the transcript assembled by Trinity, taking the longest transcript of each transcript cluster as the unigene and using it as the reference sequence for subsequent analysis.

#### 4.6.2. Functional Annotation and Analysis of Differences 

All nucleotide sequences obtained by splicing were compared, respectively, with NCBI non-redundant protein database, NCBI non-redundant nucleotide sequences database, eukaryotic orthologous groups, conserved domain database, PFAM (http://pfam.xfam.org/about), and Swissprot, TrEMBL, GO, and KEGG databases using Blast X (Version 2.2.25, E-value < 1 × 10^−5^) to obtain the corresponding annotation information. The expression levels of unigenes were calculated using transcripts per million given the effects of sequencing depth, gene length, and sample on reads. The specific equation is: 

TPM_i_ = (N_i_/L_i_) × 10^6^/sum (N_i_/L_i_ +……..+ N_m_/L_m_)

N_i_ = total exon fragment/reads 

L_i_ = exon length/KB

Differential expression analysis was performed using DESeq (Version 1.12.4) [67]. In order to obtain significantly different genes, the screening criteria were set to *q* value < 0.05 and |FoldChange| > 2. The expression pattern clustering analysis was performed on significant differential genes using the gplots Package in R (Version 2.17.0) to identify the common points of different genes with respect to expression. After the DEGs were screened, GO functional analysis and KEGG pathway analysis were performed on these DEGs. 

#### 4.6.3. qRT-PCR Analysis

All qRT-PCR primers were designed according to the mRNA CD sequence fragments obtained by transcriptome sequencing. Next, qRT-PCR was carried out with specific primers. The β-actin primer was used to detect baseline expression of mRNA in the gill tissues of different salinity groups. qRT-PCR was carried out with TaKaRa Thermal Cycler Dice^TM^ Real Time System TP800 (TaKaRa, Otsu Shiga, Japan). The reaction conditions were as follows: initial denaturation was conducted at 94 °C for 30 s; cycling at 94 °C for 5 s, 60 °C for 30 s, 40 cycles in total. The solubility curve was generated at the end of the experiment. For each RNA sample and gene, three replicates were run for all PCR analyses. mRNA levels of the target genes were calibrated using the real-time PCR *C*_t_ (2^−ΔΔ*C*t^) relative quantitative method.

### 4.7. Statistical Analysis

The statistical analysis was performed using SPSS, version 18.0 (Armonk, NY, USA). When one-way analysis of variance indicated statistical significance (*p* < 0.05), Tukey’s test was performed to examine the differences in hematological index, immune-related enzyme activity, and gene expression of *A.* *kagoshimensis* in different salinity groups. The type I error rate was controlled using the Benjamini and Hochberg procedure for multiple comparisons. Results are shown as the mean ± standard error. Sigmaplot (Systat Software Inc., San Jose, CA, USA) was operated to draw the chart against the data obtained from the said analysis.

## 5. Conclusions

In summary, in the simulation that mimicked the sudden drop of salinity in seawater after a rainstorm, we found the phagocytic activity of blood lymphocytes, the O_2_^−^ level produced from respiratory burst, the ROS content, and the activity of LZM and ACP were significantly higher in the S14 group than in the higher salinity groups. This result indicates that the immune defense mechanism of *A.* *kagoshimensis* was activated in response to salinity stress, and the non-specific immune function was enhanced by the combined action of cellular immunity and humoral immunity. Transcriptome sequencing identified 269 (101 up-regulated, 168 down-regulated), 326 (246 up-regulated, 80 down-regulated), and 185 (132 up-regulated, 53 down-regulated) significant DEGs in the comparisons of S14 vs. S22, S22 vs. S30, and S14 vs. S30, respectively. For these three comparisons, GO enrichment analysis revealed that DEGs were significantly enriched in biological process-related terms, including cellular amino acid metabolic process, regulation of protein processing, and regulation of response to stress. KEGG enrichment analysis showed that NOD-like receptor signaling pathway (ko04621), apoptosis-multiple species (ko04215), Toll and Imd signaling pathway (ko04624), NF-κB signaling pathway (ko04064), apoptosis (ko04210), and focal adhesion (ko04510) were significantly enriched in all salinity comparison groups. The qRT-PCR verification of 12 DEGs in those six pathways showed the same trend of up- and down-regulation as the results obtained from transcriptome sequencing, which confirmed that the transcriptome sequencing data were credible. 

Results of this study show that apoptosis, amino acid metabolism, and other biological processes mediated the immune regulation mechanism of *A.* *kagoshimensis* that occurred after exposure to a sudden drop of salinity. These findings suggest that the sudden drop of salinity, especially from 30‰ to 14‰, led to oxidative damage. This study provides a theoretical basis for the selection of bottom areas optimal for release and proliferation of *A.* *kagoshimensis*, as the data show that such areas must meet the condition of having a very small range of salinity variation. Otherwise, the survival rate of released *A.* *kagoshimensis* will be low. These results not only enrich the basic knowledge about the molecular biology of *A.* *kagoshimensis*, but they also provide a theoretical basis for the selection of bottom areas optimal for release and proliferation of *A.* *kagoshimensis* required to restore the declining populations of this species.

## Figures and Tables

**Table 1:** Effects of a sudden drop in salinity on total hemocyte counts, phagocytic activity, respiratory burst activity, and reactive oxygen species (ROS) content of *Anadara kagoshimensis*.

Index	Salinity Treatments			ANOVA *p*
	30‰	22‰	14‰	
Total hemocyte counts (×10^7^ cells·mL^−1^)	2.43 ± 0.19 ^b^	2.97 ± 0.31 ^a^	2.15 ± 0.24 ^b^	0.002
Phagocytic activity (%)	15.26 ± 2.26 ^c^	22.46 ± 4.17 ^b^	30.95 ± 3.55 ^a^	<0.001
Respiratory burst activity (OD_630_/10^7^ cells·mL^−1^)	0.36 ± 0.03 ^b^	0.41 ± 0.07 ^b^	0.53 ± 0.04 ^a^	0.001
Reactive Oxygen Species (A.U.)	48.21 ± 8.77 ^b^	106.38 ± 15.61 ^b^	462.19 ± 39.24 ^a^	<0.001

Values represent means and standard errors of three replicates (means ± SE; *n* = 9). Values in the same row that had different superscripts are significantly different at *p* < 0.05 based on Tukey’s test.

**Table 2:** Summary of transcriptome sequencing and assembly for *Anadara kagoshimensis*.

Items	All Number	>=500 bp Number	>=1000 bp Number	N50 Length of Unigenes (bp)	N90 Length of Unigenes (bp)	Max Length (bp)	Min Length (bp)	Total Length (bp)	Average Length (bp)
Transcript	480,426	206,812	94,603	1070	293	16,628	201	344,014,385	716.06
Unigene	194,861	71,917	30,728	933	265	16,628	201	125,490,202	644

**Table 3:** Statistics of functional annotation.

Database	Number of Genes	Percentage (%)
Annotated in CDD	22,654	11.63
Annotated in KOG	19,868	10.2
Annotated in NR	36,240	18.6
Annotated in NT	15,928	8.17
Annotated in PFAM	16,875	8.66
Annotated in Swissprot	30,091	15.44
Annotated in TrEMBL	35,539	18.24
Annotated in GO	33,522	17.2
Annotated in KEGG	12,644	6.49
Annotated in at least one database	45,883	23.55
Annotated in all database	3000	1.54
Total genes	194,861	100

CDD: conserved domain database; KOG: eukaryotic orthologous groups; NR: NCBI non-redundant protein; NT: NCBI non-redundant nucleotide sequences; GO: Gene Ontology; KEGG: Kyoto Encyclopedia of Genes and Genomes.

**Table 4:** Number of differentially expressed genes of *Anadara kagoshimensis* among different salinity treatments.

Comparison	Number of Differentially Expressed Genes		
	Up-Regulated	Down-Regulated	Total Number
S14 vs. S22	101	168	269
S22 vs. S30	246	80	326
S14 vs. S30	132	53	185

**Table 5:** Significantly enriched GO terms in DEGs at different salinity treatments.

Go No.	Go Term	Significant (n/m)	Annotated (N/M)	*p*-Value	Corrected *p*-Value
***S14* vs. *S22***					
GO:1901605	alpha-amino acid metabolic process	9/54	499/26,336	0.00000073	0.006428865
GO:0006541	glutamine metabolic process	5/54	85/26,336	0.00000087	0.006428865
GO:0006520	cellular amino acid metabolic process	10/54	728/26,336	0.000002	0.00960635
GO:1901607	alpha-amino acid biosynthetic process	6/54	191/26,336	0.0000026	0.00960635
GO:0006542	glutamine biosynthetic process	3/54	15/26,336	0.0000036	0.01064088
GO:0008652	cellular amino acid biosynthetic process	6/54	219/26,336	0.0000057	0.01404005
GO:0043436	oxoacid metabolic process	15/54	1991/26,336	0.0000073	0.015412386
GO:0006082	organic acid metabolic process	15/54	2015/26,336	0.0000085	0.015702688
GO:0035499	carnosine biosynthetic process	2/54	3/26,336	0.000012	0.019705333
GO:0019752	carboxylic acid metabolic process	14/54	1881/26,336	0.000018	0.0266022
GO:0016053	organic acid biosynthetic process	8/54	586/26,336	0.000024	0.028421154
GO:0046394	carboxylic acid biosynthetic process	8/54	586/26,336	0.000024	0.028421154
GO:0009064	glutamine family amino acid metabolic process	5/54	169/26,336	0.000025	0.028421154
GO:0006548	histidine catabolic process	2/54	5/26,336	0.000041	0.033663278
GO:0010510	regulation of acetyl-CoA biosynthetic process from pyruvate	2/54	5/26,336	0.000041	0.033663278
GO:0035498	carnosine metabolic process	2/54	5/26,336	0.000041	0.033663278
GO:0050812	regulation of acyl-CoA biosynthetic process	2/54	5/26,336	0.000041	0.033663278
GO:0052805	imidazole-containing compound catabolic process	2/54	5/26,336	0.000041	0.033663278
***S22* vs. *S30***					
GO:0070613	regulation of protein processing	7/66	118/26,336	0.000000019	0.000136706
GO:1903317	regulation of protein maturation	7/66	118/26,336	0.000000019	0.000136706
GO:0010955	negative regulation of protein processing	6/66	76/26,336	0.000000037	0.000136706
GO:1903318	negative regulation of protein maturation	6/66	76/26,336	0.000000037	0.000136706
GO:0097340	inhibition of cysteine-type endopeptidase activity	5/66	55/26,336	0.00000027	0.000570047
GO:0097341	zymogen inhibition	5/66	55/26,336	0.00000027	0.000570047
GO:1990001	inhibition of cysteine-type endopeptidase activity involved in apoptotic process	5/66	55/26,336	0.00000027	0.000570047
GO:0043154	negative regulation of cysteine-type endopeptidase activity involved in apoptotic process	5/66	125/26,336	0.000016	0.025527364
GO:0016485	protein processing	7/66	322/26,336	0.000016	0.025527364
GO:2000117	negative regulation of cysteine-type endopeptidase activity	5/66	128/26,336	0.000018	0.025527364
GO:0035499	carnosine biosynthetic process	2/66	3/26,336	0.000019	0.025527364
GO:0051604	protein maturation	7/66	349/26,336	0.000027	0.03325275
GO:0045861	negative regulation of proteolysis	6/66	245/26,336	0.000035	0.039789615
GO:0010951	negative regulation of endopeptidase activity	5/66	154/26,336	0.000043	0.043633238
GO:0010466	negative regulation of peptidase activity	5/66	157/26,336	0.000047	0.043633238
GO:0043569	negative regulation of insulin-like growth factor receptor signaling pathway	3/66	29/26,336	0.000052	0.043633238
GO:0006548	histidine catabolic process	2/66	5/26,336	0.000062	0.043633238
GO:0010510	regulation of acetyl-CoA biosynthetic process from pyruvate	2/66	5/26,336	0.000062	0.043633238
GO:0035498	carnosine metabolic process	2/66	5/26,336	0.000062	0.043633238
GO:0050812	regulation of acyl-CoA biosynthetic process	2/66	5/26,336	0.000062	0.043633238
GO:0052805	imidazole-containing compound catabolic process	2/66	5/26,336	0.000062	0.043633238

M is the number of all the genes with GO annotation; m is the number of DEGs in M; N is the number of all the genes that are annotated to the certain GO terms; n is the number of DEGs in N.

**Table 6:** Oligonucleotide primers designed for qRT-PCR of twelve candidate DEGs.

Unigene-ID	Gene Name	Sequence (5’-3’)	Efficiency	Product Size (bp)
TRINITY_DN64699_c0_g1	*IAP*	F: GCAAACTTCTTGCCGTGCGGCA	97.80%	168
		R: CTGCATGCGACACCCAATCAG		
TRINITY_DN65676_c2_g1	*BIRC3*	F: ACGGACCAGGCAGTTGTCGT	102.43%	192
		R: CGCCGGTTGTGTGGCCACCCTGT		
TRINITY_DN65035_c3_g1	*GLUL*	F: GAATCTTCTCGCAAATCCGCG	98.15%	177
		R: ATTGATTGTTGTGCTTCCC		
TRINITY_DN53170_c0_g1	*GADD45G*	F: ACCGCCGAACCAGTATTTGGC	107.81%	204
		R: CAGGAAACACGGAGCCGATG		
TRINITY_DN63352_c4_g2	*CECR5*	F: AGTTTGACCTCCTGCTGACC	101.92%	225
		R: TGACATCTTGAAATCCGGGCA		
TRINITY_DN57342_c5_g1	*CHKB*	F: CGCAACCTGCCTAAGTCTCT	98.75%	159
		R: GGTCCGCTACGTTGTGACCGC		
TRINITY_DN41276_c0_g1	*TKTL1*	F: CTTCTAACTGCTCCTTCGGAATTC	105.38%	207
		R: GAACACACCACGGCACTAAGCC		
TRINITY_DN63081_c2_g1	*UCP2*	F: CAGATAACATCAGTTCTGGACG	96.75%	190
		R: ACCGGTCAATGTTCCAGTCCTT		
TRINITY_DN72946_c3_g5	*CYP2G1*	F: ATCCTCTTGGACATCACCGA	100.37%	146
		R: GGAACAGGACCAGGCAGAAG		
TRINITY_DN59014_c0_g1	*CARNS1*	F: GACCGGTGATGAAGAATTG	99.26%	188
		R: GTTAAAGCCTAGCAGATT		
TRINITY_DN66154_c0_g2	*GRP78*	F: ACACCTCCGACGAAATATTCC	101.79%	201
		R: CATCAACCGGGACACGATCGCC		
TRINITY_DN64918_c2_g2	*RPL39*	F: CACTTCAATCGCCTGGTTCAAT	98.59%	173
		R: GACCCCTGGTATTTGTGCCAG		

F: forward primer; R: reverse primer.

## Supplementary Materials

Supplementary materials can be found at https://www.mdpi.com/1422-0067/20/18/4365/s1.
