# Supplementary material for: Effects of a Sudden Drop in Salinity on Immune Response Mechanisms of Anadara kagoshimensis
Source: Int J Mol Sci. 2019 Sep 5;20(18):4365. doi: 10.3390/ijms20184365 (PMC6769905; doi:10.3390/ijms20184365)
Supplement: Supplementary file 1 [file ijms-20-04365-s001.pdf]

A

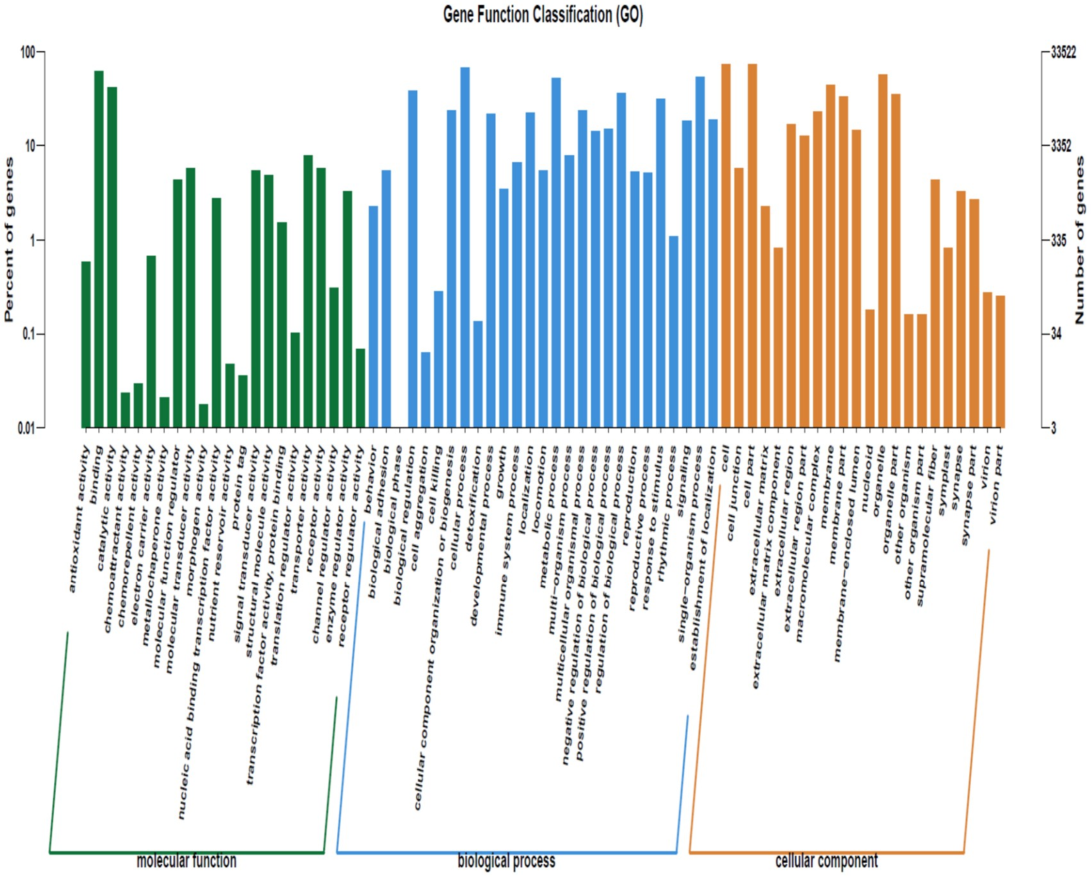
Gene Function Classification (GO)
Percent of genes
Number of genes
100
10
1
0.1
0.01
33522
3352
335
34
3
antioxidant activity
binding
catalytic activity
chemoattractant activity
chemorepellent activity
electron carrier activity
metallochaperone activity
molecular function regulator
molecular transducer activity
morphogen activity
nucleic acid binding transcription factor activity
nutrient reservoir activity
protein tag
signal transducer activity
structural molecule activity
transcription factor activity, protein binding
translation regulator activity
transporter activity
receptor activity
channel regulator activity
enzyme regulator activity
receptor regulator activity
behavior
biological adhesion
biological phase
biological regulation
cell aggregation
cell killing
cellular component organization or biogenesis
cellular process
detoxification
developmental process
growth
immune system process
localization
locomotion
metabolic process
multi−organism process
multicellular organismal process
negative regulation of biological process
positive regulation of biological process
regulation of biological process
reproduction
reproductive process
response to stimulus
rhythmic process
signaling
single−organism process
establishment of localization
cell
cell junction
cell part
extracellular matrix
extracellular matrix component
extracellular region
extracellular region part
macromolecular complex
membrane
membrane part
membrane−enclosed lumen
nucleoid
organelle
organelle part
other organism
other organism part
supramolecular fiber
symplast
synapse
synapse part
virion
virion part
molecular function
biological process
cellular component

B

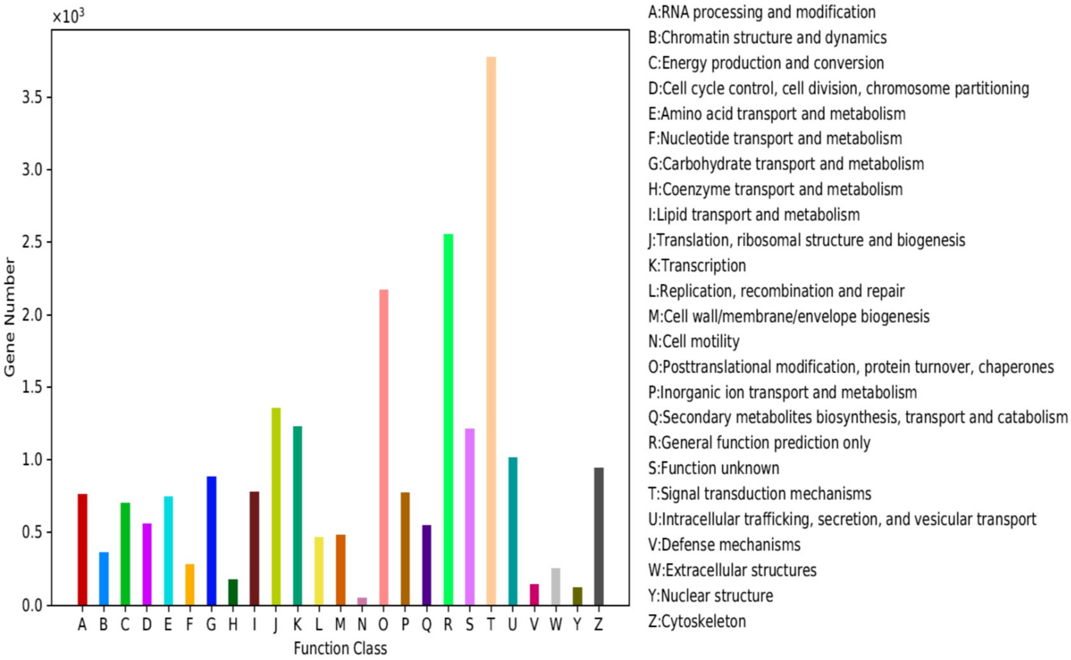
×10^{3}
Gene Number
3.5
3.0
2.5
2.0
1.5
1.0
0.5
0.0
A B C D E F G H I J K L M N O P Q R S T U V W Y Z
Function Class
A:RNA processing and modification
B:Chromatin structure and dynamics
C:Energy production and conversion
D:Cell cycle control, cell division, chromosome partitioning
E:Amino acid transport and metabolism
F:Nucleotide transport and metabolism
G:Carbohydrate transport and metabolism
H:Coenzyme transport and metabolism
I:Lipid transport and metabolism
J:Translation, ribosomal structure and biogenesis
K:Transcription
L:Replication, recombination and repair
M:Cell wall/membrane/envelope biogenesis
N:Cell motility
O:Posttranslational modification, protein turnover, chaperones
P:Inorganic ion transport and metabolism
Q:Secondary metabolites biosynthesis, transport and catabolism
R:General function prediction only
S:Function unknown
T:Signal transduction mechanisms
U:Intracellular trafficking, secretion, and vesicular transport
V:Defense mechanisms
W:Extracellular structures
Y:Nuclear structure
Z:Cytoskeleton

C

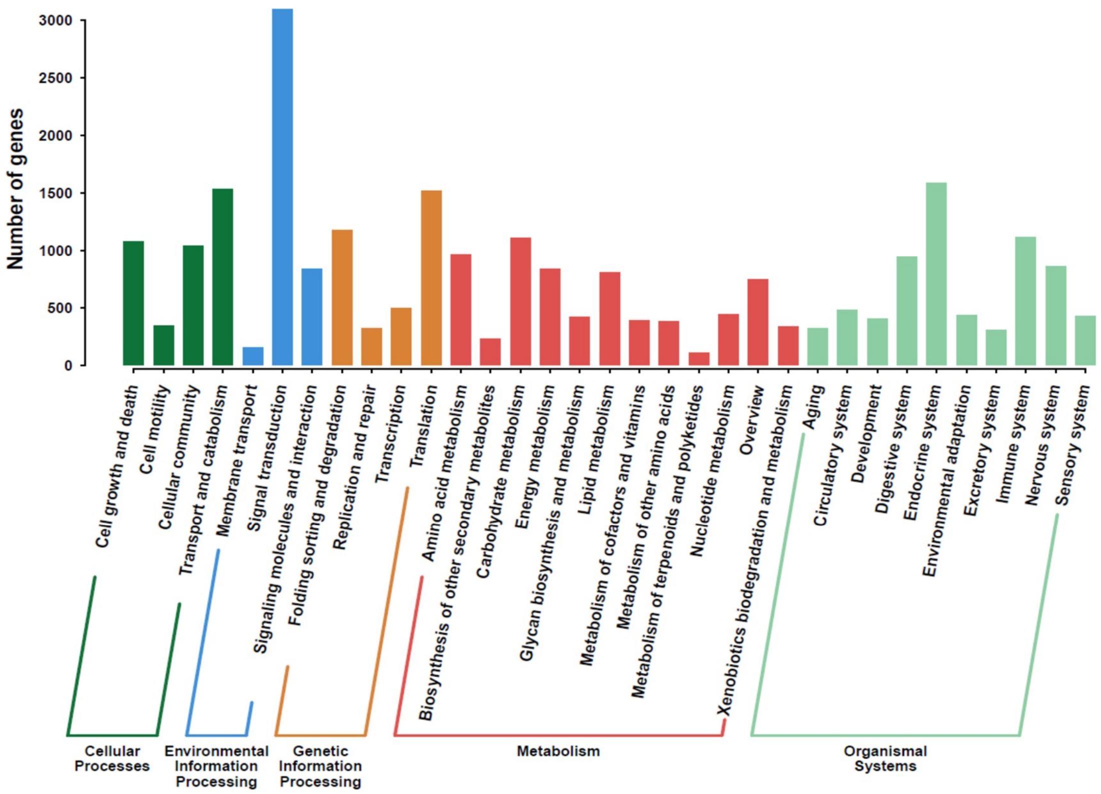

Figure S1. Similarity analysis and gene function annotation in *Anadara kagoshimensis*. A: GO (Gene ontology) classification of the assembled unigenes; B: KOG (EuKaryotic Orthologous Groups) classification of the assembled unigenes; C: KEGG (Kyoto Encyclopedia of Genes and Genomes) annotation analysis of the assembled unigenes.

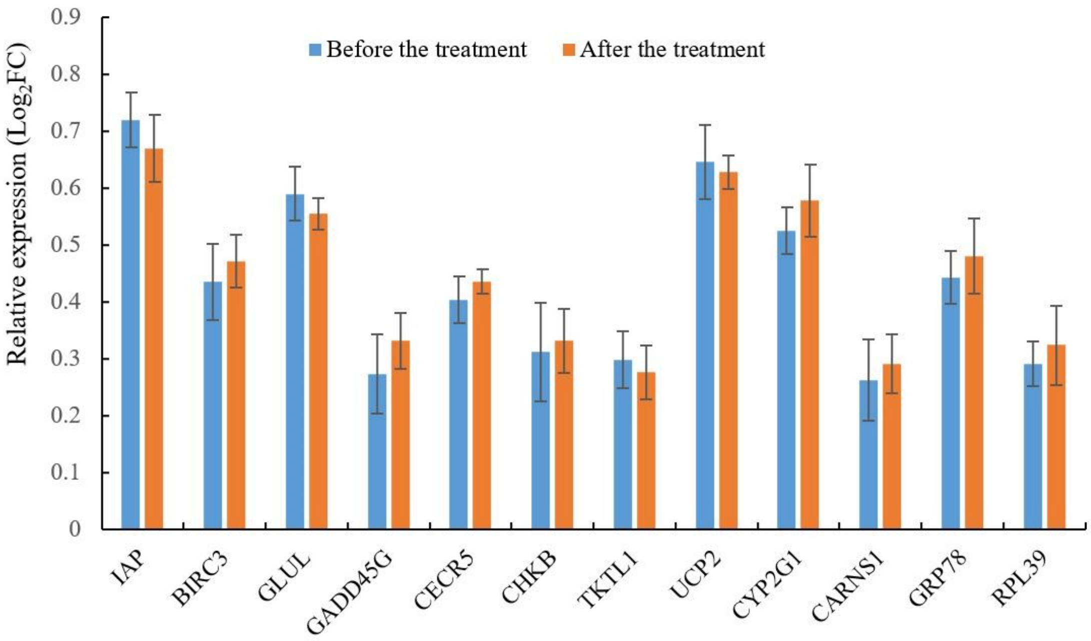

Figure S2. QRT-PCR analysis for 12 DEGs in *Anadara kagoshimensis* before and after 30‰ salinity treatment. The relative expression values were normalized to β-actin gene expression. Vertical bars represent the mean ± S.D. (n = 3).
